# Deletion of TXNDC5 downregulates TGFβ1-αSMA-mediated testicular fibrosis in mice

**DOI:** 10.1530/REP-25-0022

**Published:** 2025-05-02

**Authors:** Ya-Yi Yang, Yi-Min Chiang, Kai-Chien Yang, Pei-Shiue Tsai

**Affiliations:** ^1^ Department of Veterinary Medicine, National Taiwan University, Taipei, Taiwan; ^2^ Graduate Institute of Veterinary Medicine, National Taiwan University, Taipei, Taiwan; ^3^ Department of Surgery, National Taiwan University Hospital, Taipei, Taiwan; ^4^ Department and Graduate Institute of Pharmacology, National Taiwan University College of Medicine, Taipei, Taiwan; ^5^ Research Center for Developmental Biology and Regenerative Medicine, National Taiwan University, Taipei, Taiwan; ^6^ Division of Cardiology, Department of Internal Medicine, National Taiwan University Hospital, Taipei, Taiwan; ^7^ Institute of Biomedical Sciences, Academia Sinica, Taipei, Taiwan

**Keywords:** fibrosis, ischemia/reperfusion, testicular torsion, TXNDC5

## Abstract

**In brief:**

Testicular fibrosis is a common complication in testicular torsion cases, resulting in infertility. This paper reveals the alleviated role of an ER protein TXNDC5 in ischemia/reperfusion-induced testicular fibrosis mouse model, providing its therapeutic potential.

**Abstract:**

Testicular torsion is a urological emergency. Delayed diagnosis and treatment hamper the testicular salvage rate and the subsequent recovery of the fertility. Although detorsion surgery restores the testicular blood flow, the process of torsion/detorsion leads to ischemia/reperfusion injury, which aggravates the primary damage due to the excessive generation of free radicals that disrupt antioxidant homeostasis. In the present study, we investigated the role of endoplasmic reticulum protein, thioredoxin domain-containing protein 5, in ischemia/reperfusion-induced testicular fibrosis. We showed that apart from disrupted testicular structure, elevated transforming growth factor beta 1, alpha-smooth muscle actin and collagen type 1 protein expression accompanied by TXNDC5 upregulation were detected in mice that underwent ischemia/reperfusion injury. By *Txndc5* deletion, fibrosis, redox oxidation and extracellular matrix-associated signaling pathways and their accompanied genes/proteins were downregulated, indicating amelioration of testicular tissue damage and fibrosis in *Txndc5^−/−^* mice. With the apparent restoration of testicular structure and spermatogenesis, our study provides a potential therapeutic strategy by targeting TXNDC5 in testicular torsion patients to reduce ischemia/reperfusion-induced testicular fibrosis and to restore their fertility.

## Introduction

Testicular torsion is a urological emergency, in which the rotated testis and twisted spermatic cord lead to the obstruction of blood circulation toward the testis (Lacy *et al.* 2023). The incidence rate of testicular torsion is 1 in 4,000 males less than 25-years-old, with higher occurrence in neonates and post-pubertal patients (Oliveira *et al.* 2024). Despite an immediate detorsion surgery, testicular atrophy, testicular fibrosis, permanent testicular dysfunction (Reddy *et al.* 2023) and infertility were often observed in patients in the postoperative follow-up (Emad-Eldin *et al.* 2015, Yildirim *et al.* 2018, Azılı *et al.* 2021, Ho *et al.* 2021, Guazzone & Lustig 2023). The pathophysiology of testicular torsion centers on ischemia/reperfusion (IR) injury, in which the twisted spermatic cord results in testicular tissue ischemia, followed by reperfusion resulting from the releasing of the twisted spermatic cord. During the ischemic period, the testicular tissue becomes hypoxia; a low level of oxygen depletes energy sources, leads to a decrease in ATP production, and restricts the uptake of calcium by the endoplasmic reticulum (ER). Intracellular calcium overload and accumulation of cellular metabolites subsequently activate apoptotic responses in testicular cells. Despite reperfusion restoring the delivery of oxygen required for aerobic metabolism, the re-establishment of blood supply after prolonged ischemia boosts reactive oxygen species (ROS) production by reintroducing oxygen molecules as the fuel for redox reaction. The overloaded free radicals cause oxidative stress, DNA damage, protein dysfunction and lipid peroxidation, further disrupting the membrane permeability, potential and integrity of mitochondria. ROS derived from IR injury was also involved in generating proinflammatory cytokines, recruitment of circulating leukocytes to the testis and alteration of ion transport that terminally results in germ cell loss and subfertility/infertility (Kalogeris *et al.* 2012, Karaguzel *et al.* 2014, Yildirim *et al.* 2018).

Our protein of interest, thioredoxin domain-containing protein 5 (TXNDC5), is an ER resident protein that belongs to the protein disulfide isomerase (PDI) family. It is also known as ER protein 46 (Erp46) (Knoblach *et al.* 2003), endothelial protein disulfide isomerase (EndoPDI) (Sullivan *et al.* 2003) or protein disulfide isomerase family A, member 15 (PDIA15), with a molecular weight of 48 kDa. First identified in hypoxic tissues with enhanced activity (Wang *et al.* 2022), TXNDC5 regulates protein folding by facilitating the formation and rearrangement of disulfide bonds through its thioredoxin (TRX) domains (Horna-Terrón *et al.* 2014). While TXNDC5 dysregulation has been shown in multiple diseases, such as sepsis, rheumatoid arthritis, diabetes and cancers, by regulating inflammatory factors (Jiao *et al.* 2024), emerging evidence demonstrated that TXNDC5 acts as a key pathogenic factor in organ fibrosis, including heart (Shih *et al.* 2018), lung (Lee *et al.* 2020), kidney (Chen *et al.* 2021) and liver (Hung *et al.* 2022*a*). Earlier studies showed that TXNDC5 was upregulated in collagen-secreting fibroblasts and regulated the TGFβ1-induced fibrogenic signaling pathway. TXNDC5 promotes fibrosis by mediating the proper folding of extracellular matrix (ECM) proteins such as collagen, stabilizing TGFβ receptor one protein and activating the proliferation of fibroblasts. Hence, increased TXNDC5 expression facilitates the development of organ fibrosis by promoting myofibroblasts activation and enhancing excessive ECM protein deposition (Hung *et al.* 2022*b*).

Although TXNDC5 is crucial in cardiac, pulmonary, renal and liver fibrosis, instead of enriched epithelium and mesothelium in the abovementioned organs, the testis is mainly composed of spermatogenic cells, Leydig cells, Sertoli cells and peritubular myoid cells with few testicular fibroblasts (Ma *et al.* 2023). The distinct and complex cellular compositions, rapid and constant proliferation characteristics of the testis likely render its unique organ feature in the progression of fibrosis and its repair mechanism. Therefore, whether TXNDC5 plays a role in regulating testicular fibrosis remains unresolved. In this study, we aim to reveal the necessity of TXNDC5 in ischemia/reperfusion (IR)-induced testicular fibrosis and explore the underlying regulatory mechanism.

## Materials and methods

### Chemicals, reagents and antibodies

All chemicals and reagents were purchased from Sigma Aldrich (USA), unless otherwise stated. Rabbit polyclonal anti-transforming growth factor beta 1 (TGFβ1, #Ab92486), goat polyclonal anti-alpha smooth muscle actin (αSMA, #Ab21027), mouse monoclonal anti-collagen type 1 (COL1, #Ab6308) and rabbit monoclonal anti-eukaryotic elongation factor 2 (EEF2, #Ab75748) were purchased from Abcam (UK). Rabbit polyclonal anti-thioredoxin domain-containing 5 (TXNDC5, #19834-1-AP) was obtained from Proteintech (USA). All secondary antibodies were purchased from Jackson ImmunoResearch Laboratories Inc. (USA).

### Animals

Male C57B6/J mice were obtained from BioLASCO, Taiwan Co., Ltd (Taiwan) and monitored daily by certified veterinarians. All mice were accommodated at a constant temperature (22–24°C) with a 12 h light:12h darkness cycle, clean water and standard laboratory chow (Oriental Yeast, Japan) were given *ad libitum*. Animal experiments were carried out under the permission and surveillance of institutional animal care and use committee (IACUC) protocols (NTU-109-EL-00170, NTU-110-EL-00147) at National Taiwan University. To establish the ischemia/reperfusion-induced testicular fibrosis mouse model, 15-week-old male C57BL/6J (wildtype) mice were used. For the evaluation of the role of TXNDC5 in IR-induced testicular fibrosis, *Txndc5^−/−^* mice, kindly provided by Professor Kai-Chien Yang from National Taiwan University Hospital, were used.

### Establishment of the ischemia/reperfusion-induced testicular fibrosis mouse model

General anesthesia was achieved by inhalation of 1∼3% isoflurane throughout the following surgical procedures (Supplementary Fig. 1A (see section on Supplementary materials given at the end of the article)). The lower abdominal area of the mouse was shaved, and the skin was disinfected with 75% ethanol solution. A horizontal incision was made on the peritoneal cavity to expose bilateral testes from the scrotum. In the sham surgical group, the gubernaculum of both testes was cut off to disconnect the testes from the scrotum. After placing the testes back into the scrotum, the skin was sutured immediately without torsion and detorsion procedures. In the ischemia/reperfusion (IR) group, testicular ischemia was created by rotating the testes 720° clockwise with the testicular arteries clamped by sterilized artery clips for 1 h (Supplementary Fig. 1A, B, C). The operating field was covered with gauze immersed in 0.9% normal saline to prevent tissues from drying. After the first ischemic procedure, the artery clips were removed, and testes were temporarily twisted 720° counterclockwise to their original orientation for 15 min, followed by rotating 720° clockwise with the vasculature blocked by artery clips again for another 1 h. After the second ischemic procedure, the artery clips were removed, and testes were twisted 720° counterclockwise for final reperfusion (Supplementary Fig. 1A and D). Restoration of the testicular blood flow was monitored for 5 min before the final suture procedures. The muscle layer of the abdominal incision was closed by sterile absorbable Monosyn® 5-0 suture (B. BRAUN Surgical, S.A., Spain) with a simple continuous pattern, and the cutaneous layer was closed by simple interrupt sutures using the Coated VICRYL® 3-0 suture (Ethicon, Inc., USA) (Supplementary Fig. 1A and E). Antibiotics (cefazolin, 20 mg/kg B.W., Standard Chem & Pharm CO., LTD, Taiwan) were given via intramuscular injection upon mice recovery from anesthesia. Mice were monitored daily for general physical conditions and wound healing. Six weeks after the surgery, mice were euthanized with CO_2,_ followed by cervical dislocation (Supplementary Fig. 1A).

### Histological evaluations of the testes

Testicular weight and size were measured at the end of the sixth week before being processed for histological evaluations. One side of the testes was fixed in 10% neutral buffered formalin overnight on the shaker, while the other side of the testes was flash-frozen in liquid nitrogen and stored at −80°C for further use. Formalin-fixed testes were processed with standard paraffin-embedding procedures and sectioned in 5 μm thickness. For staining purposes, slides were deparaffinized in 100% xylene and sequentially rehydrated in 100∼80% ethanol. Sections were stained with hematoxylin and eosin (H&E) for general histopathological evaluation and Masson’s trichrome for observation of collagen deposition. All testicular sections were examined using the Olympus IX83 microscopy. Under the H&E stain, the severity of testicular damage was graded by the Johnsen scoring system from 10 to 1 point according to its histological criteria (Table 1) (Johnsen 1970). A lower point represents a more severe loss of germinal cells in the seminiferous tubule. For each individual, total points were added up and divided by the number of lumens evaluated to obtain the average score per lumen. In Masson’s trichrome stain, the collagen-positive area was quantified using the ImageJ software. The color threshold (the hue was ‘145–200’, the saturation was ‘90–255’ and the brightness was ‘100–255’) was set up to achieve optimal blue color measurement (Supplementary 2A, B, C). Selected blue positive areas were divided by the total area of each image (%), and the average value was calculated for each individual (24 random images total in each experimental group) (Supplementary 2D and F).

**Table 1:** Johnsen scoring system for evaluating testicular damage.

Johnsen score	Description of histological criteria
10	Full spermatogenesis
9	Slightly impaired spermatogenesis, many late spermatids, disorganized epithelium
8	Less than five spermatozoa per tubule, few late spermatids
7	No spermatozoa, no late spermatids, many early spermatids
6	No spermatozoa, no late spermatids, few early spermatids
5	No spermatozoa or spermatids, many spermatocytes
4	No spermatozoa or spermatids, few spermatocytes
3	Spermatogonia only
2	No germinal cells, sertoli cells only
1	No seminiferous epithelium

### Polymerase chain reaction (PCR)-based genotyping of *Txndc5^−/−^* transgenic mice

*Txndc5^−/−^* mice were generated at the Transgenic Mouse Core Laboratory at National Taiwan University, and were given by Prof. KC Yang as research collaboration materials. Genetic background validations were processed by PCR (Supplementary Fig. 3A). In short, the toes of mice were obtained and heated in 300 μL NaOH (50 mM) at 100°C for 1 h for genomic DNA extraction. The PCR mixture was prepared by mixing 1 μL DNA extract, 10 μL amaR OnePCR^TM^ (GeneDireX, Inc., Taiwan) and 0.4 μL 10 μM forward and reverse primer sets (Supplementary Fig. 3B). The PCR program is illustrated in Fig. S3C; agarose gel electrophoresis was used for DNA separation, and results were visualized by the UV transilluminator (Analytik Jena US LLC, USA).

### Indirect immunofluorescence (IFA) staining

Paraffin-embedded tissue sections were deparaffinized in 100% xylene and rehydrated in 100∼80% ethanol, as mentioned above. Antigen retrieval was performed with 10 mM citrate buffer (pH 9.0) by microwaving for 10 min at 105°C. After the citrate buffer cooled down, tissue sections were permeabilized with 100% methanol for 10 min at −20°C. Nonspecific signals were blocked by 5% bovine serum albumin (BSA, in filtered PBS) for 1 h at room temperature. Primary antibodies including anti-TGFβ1, anti-αSMA, anti-COL1 and anti-TXNDC5 were diluted in 1:200, 1:200, 1:100 and 1:100 ratio, respectively, and tissue sections were incubated overnight at 4°C. After washing with PBS, secondary antibodies including donkey anti-rabbit Alexa 594, donkey anti-goat Alexa 488 and donkey anti-mouse Alexa 594 (1:150 dilution in 5% BSA) were subsequently applied for 1.5 h at room temperature. Cell nuclei were counterstained with 4′, 6-diamidino-2-phenylindole (DAPI) (Vectashield H-1200, Vector Laboratories, UK) and sections were sealed with transparent nail polish. As for negative controls, slides were processed as mentioned above but omitted the incubation of the primary antibody. All samples were examined with the Olympus IX83 epifluorescence microscopy.

### Immunoblotting

Frozen testes were homogenized with homogenizing buffer (250 mM sucrose, 1 mM EDTA, 20 mM, 1% Triton X-100, pH 7.5) supplemented with 1X protease inhibitor cocktail (Roche, Switzerland; 11836153001, 4°C) and protein concentration was quantified with BCA quantification assay (Thermo Fisher Scientific, USA). Equivalent quantities (μg) of protein extracts were mixed with lithium dodecyl sulfate sample buffer (NuPAGE^TM^, Thermo Fisher Scientific) and 50 mM dithiothreitol (DTT). Samples were denatured for 10 min at 100°C and cooled down to room temperature by vortexing. For electrophoresis, a Bio-Rad Mini-PROTEIN® electrophoresis system (Bio-Rad Laboratories LTD, UK) was used. Proteins were separated by a 9% sodium dodecyl sulfate-polyacrylamide gel (SDS-PAGE) and wet-blotted onto a polyvinylidene difluoride (PVDF) membrane (Immobilon-P, Millipore, USA). The PVDF membrane was subsequently blocked with blocking buffer (5% milk powder in TBST (5 mM Tris, 250 mM sucrose, pH 7.4 with 0.05% v/v Tween-20)) for 1 h at room temperature before being incubated with anti-TGFβ1 antibody (1:1,000 dilution), anti-αSMA antibody (1:1,000 dilution), anti-COL1 antibody (1:5,000 dilution), anti-TXNDC5 antibody (1:1,000 dilution) or anti-EEF2 antibody (1:50,000 dilution) overnight at 4°C. After washing with TBST, secondary antibodies including anti-rabbit HRP, anti-goat HRP or anti-mouse HRP (1:10,000 dilution in blocking buffer) were applied for another 1 h. Protein signals were detected by chemiluminescence (Merck, Ltd, USA) and visualized under the ChemiDoc XRS+ System (Bio-Rad Laboratories, USA). The intensity of each band was semi-quantified by the ImageJ software. To detect other proteins of interest, the stripping buffer (Thermo Fisher Scientific) was used before reprobing the membrane with other antibodies.

### Next-generation sequencing (NGS)

Testes from wildtype control (*Txndc5^+/+^*, *n* = 3), wildtype IR-treated (*n* = 3), *TXNDC5* knockout control (*Txndc5^−/−^*, *n* = 3) and *Txndc5* knockout IR-treated (*n* = 3) mice were harvested for next-generation sequencing and bioinformatics analysis. Total testicular RNA extracts were subjected to cDNA synthesis and NGS library construction using the Universal Plus mRNA-Seq Library Preparation Kit (TECAN, USA). The quality and average length of the sequence library for each sample were evaluated by the bioanalyzer and the DNA 1000 kit (Aligent Technologies, USA). The indexed samples were pooled equimolarly on the Illumina NovaSeq 6000 platform (Illumina, USA) and underwent high-throughput sequencing. Raw reads were quantified by the CLC Genomics Workbench v.10 software (QIAGEN, The Netherlands). Low-quality sequences, ambiguous nucleotides and adapter sequences were trimmed. The trimmed reads were mapped to the *Mus musculus* genome GRCm38 (mm10) assembly from the C57BL/6J strain using the CLC Genomics Workbench. The mapping parameters were as follows: mismatch cost 2, insertion cost 3, deletion cost 3, length fraction of 0.5 and similarity fraction of 0.8. The differentially expressed genes (DEGs) between two or more groups were based on the fold change of FPKM (fragments per kilobases per million) value, and *P*-values were adjusted by the Benjamini–Hochberg’s FDR correction. Genes with FDR corrected *P*-value <0.05 were considered to be significantly differentially expressed and only genes with a two-fold change were further examined for downstream analysis. KEGG database (Kanehisa & Goto 2000, Kanehisa *et al.* 2016) was used in pathway enrichment analysis. Modified Fisher exact *P*-value (EASE score) was used and KEGG pathways with *P*-value <0.05 were considered to be highly associated with respective comparing groups. Gene ontology (GO) enrichment was analyzed by GO-TermFinder (Boyle *et al.* 2004).

### Statistical analysis

All values are presented as the mean ± standard deviation (SD). One-way analysis of variance (ANOVA) with Tukey’s multiple comparisons test or Šídák’s multiple comparisons test were carried out with the GraphPad Prism (GraphPad Software, USA) for statistical analyses. Before one-way ANOVA analysis, Brown–Forsythe test was used to validate the assumption of normality and homogeneity of variances. Statistical significances were considered when the *P*-value was <0.05.

## Results

### Repeated IR injury-induced severe testicular fibrosis

**Figure 1:**
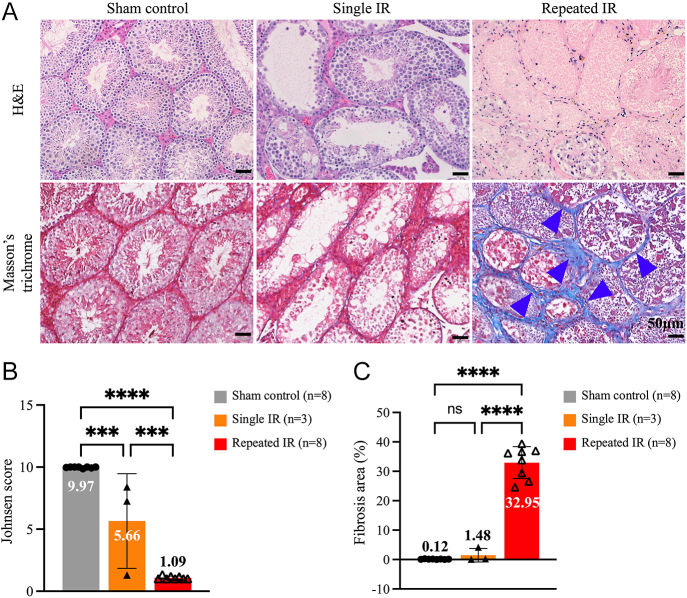
Histological evaluation of testicular damage after IR injuries. (A) H&E and Masson’s trichrome staining of sham control, single IR and repeated IR testes. Necrotic changes of testicular cells were only observed in some lumens after the single IR procedure, but repeated IR testes showed a total loss of germinal cell layers with cell debris left in the seminiferous tubules. Under Masson’s trichrome stain, blue-stained collagen fibers were abundant in the repeated IR testes (indicated by arrowheads). (B) Testicular damage was quantitatively assessed by the Johnsen scoring system. (C) A significant increase in fibrosis area (%) in repeated IR-treated testes demonstrated the success of our IR-induced testicular fibrosis mouse model. One-way analysis of variance (ANOVA) with Šídák’s multiple comparisons test was used for group comparisons and statistical difference at *P* < 0.05 (****P < 0.0001, ***P < 0.001, ns not statistically different). IR, ischemia/reperfusion.

To establish a reproducible IR-induced testicular fibrosis mouse model, we performed single IR and repeated IR procedures on the testes. To validate the success of our mouse model, histological evaluation and Johnsen score (Johnsen 1970) were used. In single IR, testes were characterized with signs of apoptosis and necrosis, such as hydropic degeneration and pyknosis, with decreased layers of spermatogenic cells in some lumens (Fig. 1A, middle panel). When IR injury was repeated, testes showed complete loss of spermatogenic cell layers and destroyed histological architecture of seminiferous tubules, with excessive collagen fibers accumulated in the interstitial area, representing the formation of fibrosis (Fig. 1A, right panel). Repeated IR-treated testes were scored significantly lower (Fig. 1B, red bar) in the Johnsen scoring system when compared to sham control (Fig. 1B, gray bar) and single IR (Fig. 1B, orange bar) groups, demonstrating aggravated damages to the testes. Although single IR-treated testes had significantly lower scores when compared to sham control, large individual variations were observed (Fig. 1B). Besides apparent damage on the seminiferous tubules, compared to sham control (Fig. 1C, gray bar) and single IR (Fig. 1C, orange bar) groups, the percentage of fibrosis area increased significantly after repeated IR injury (Fig. 1C, red bar) demonstrated the success of our model.

### TGFβ, αSMA and COL1 were upregulated in IR-induced testes

**Figure 2:**
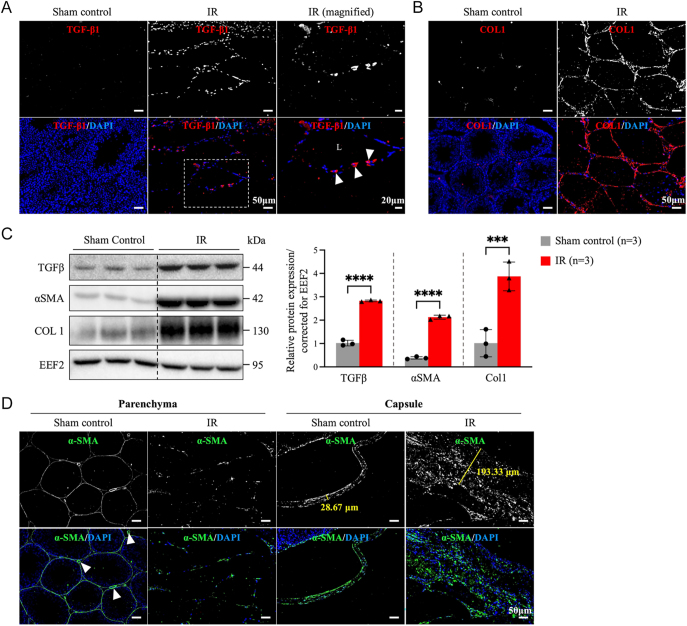
The effects of IR injury on the expression of fibrosis-relevant proteins. (A) Upon IR-injury, TGFβ was upregulated in the cells located around the lumens (indicated with arrowheads). (B) Excessive amount of type I collagen accumulation in the interstitial region of the IR testes. (C) Western blotting analyses showed that IR promoted TGFβ, αSMA and COL1 protein expression in the testes. (D) In the parenchyma of the control testes, αSMA showed a continuous staining pattern around the seminiferous tubules and blood vessels (arrowheads); however, the signals became discontinuous in IR testes. Apart from parenchyma, the upregulation of αSMA in the capsule was also observed when compared to the control (indicated with yellow lines). One-way analysis of variance (ANOVA) with Tukey’s multiple comparisons test was used for group comparisons and statistical difference at *P* < 0.05 (****P < 0.0001, ***P < 0.001). L, lumen.

Transforming growth factor-β (TGFβ), the master regulator of fibrosis, is a well-known factor that triggers fibrosis in various organs in response to tissue injury (Lurje *et al.* 2023). To investigate the cellular and molecular mechanisms of IR-induced testicular fibrosis, immunofluorescence staining and immunoblotting of TGFβ, downstream α-smooth muscle actin (αSMA) and collagen type 1 (COL1) were performed. Increased TGFβ signals were observed around the seminiferous tubules in IR-treated testis (Fig. 2A, indicated with arrowheads). Accumulation of COL1 was also detected in the interstitial region of the testis (Fig. 2B), which correlated to the localization of blue-stained collagen fibers shown in Masson’s trichrome stain (Fig. 1A). Quantitative analysis confirmed that the overall protein expression level of TGFβ, αSMA and COL1 enhanced significantly in the IR-injured testes (Fig. 2C). In sham control testis, besides blood vessels (served as positive control, Fig. 2D, indicated with arrowheads), αSMA lined around the seminiferous tubules where peritubular cells are located. Interestingly, although increased αSMA signals were detected in the thickened capsule, indicating increased myofibroblast differentiation at the testicular capsule (Fig. 2D, indicated with yellow lines), the signals of αSMA in the parenchyma area of the testis decreased significantly after IR injury (Fig. 2D).

### Deletion of *Txndc5* significantly alleviated IR-induced testicular fibrosis

**Figure 3:**
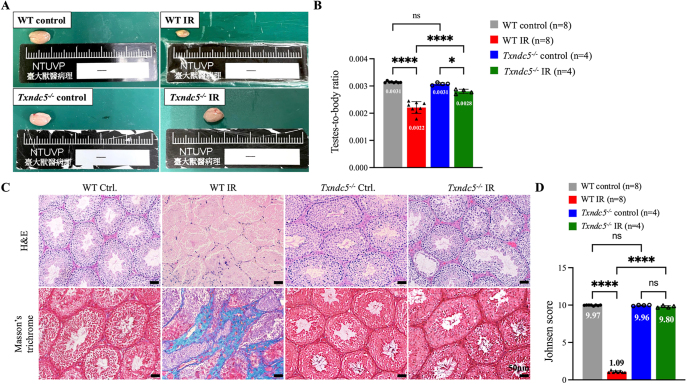
The effects of *Txndc5* KO on IR-induced testicular damages. (A) In contrast to WT control, WT IR testis was smaller in size with a firm texture. A recovery in size was observed in *Txndc5^−/−^* IR testes with normal soft texture. (B) The testes-to-body ratio decreased significantly in WT IR testes compared to the WT control group. Although *Txndc5^−/−^* IR testes also showed decreased testes-to-body ratio compared to *Txndc5^−/−^* control, a significant increase was detected when comparing *Txndc5^−/−^* IR to WT IR group, suggesting a recovery in testicular weight. (C) Restoration of spermatogenic cell layers (H&E) and decreased collagen deposition (Masson’s trichrome) were observed in *Txndc5^−/−^* IR testicular sections. (D) In contrast to WT mice, the Johnsen scoring system showed no significant differences between the *Txndc5^−/−^* IR group and the *Txndc5^−/−^* control group, indicating decreased testicular damage after knocking out *Txndc5*. One-way analysis of variance (ANOVA) with Tukey’s multiple comparisons test was used for group comparisons and statistical difference at *P* < 0.05 (****P < 0.0001, *P < 0.05, ns not statistically different).

The effects of *TXNDC5* in IR-induced testicular fibrosis were first assessed by gross examination and histopathological evaluations. In WT mice, the size and the texture of IR testes were small and firm when compared to control testes (Fig. 3A, upper panel). In a sharp contrast, the testicular size and the texture of the *Txndc5^−/−^* mice remained normal and soft (Fig. 3A, lower panel). Although a decrease in the testes-to-body ratio was still detected in the *Txndc5^−/−^* mice when compared with their non-IR control, a marked recovery was measured in the *Txndc5^−/−^* IR group when compared with WT-IR testes, indicating a significant restoration of testicular weight in *Txndc5^−/−^* mice (Fig. 3B). Unlike WT IR testis showed loss of all spermatogenic cell layers and disrupted testicular architecture, *Txndc5^−/−^* IR testis exhibited normal testicular structure, with more than four spermatogenic layers in most seminiferous tubules (Fig. 3C, H&E). Masson’s trichrome stain revealed a decrease in collagen deposition in *Txndc5^−/−^* IR testis compared to WT IR testis, indicating that *Txndc5* knockout not only restored the testicular structure but also alleviated testicular fibrosis (Fig. 3C). The Johnsen scoring system further validated the significant recovery of testicular damage from IR injury in *Txndc5^−/−^* testes (Fig. 3D, green bar).

### IR injury significantly increased testicular TXNDC5 protein expression and *Txndc5* KO markedly reduced fibrosis-related protein expressions

**Figure 4:**
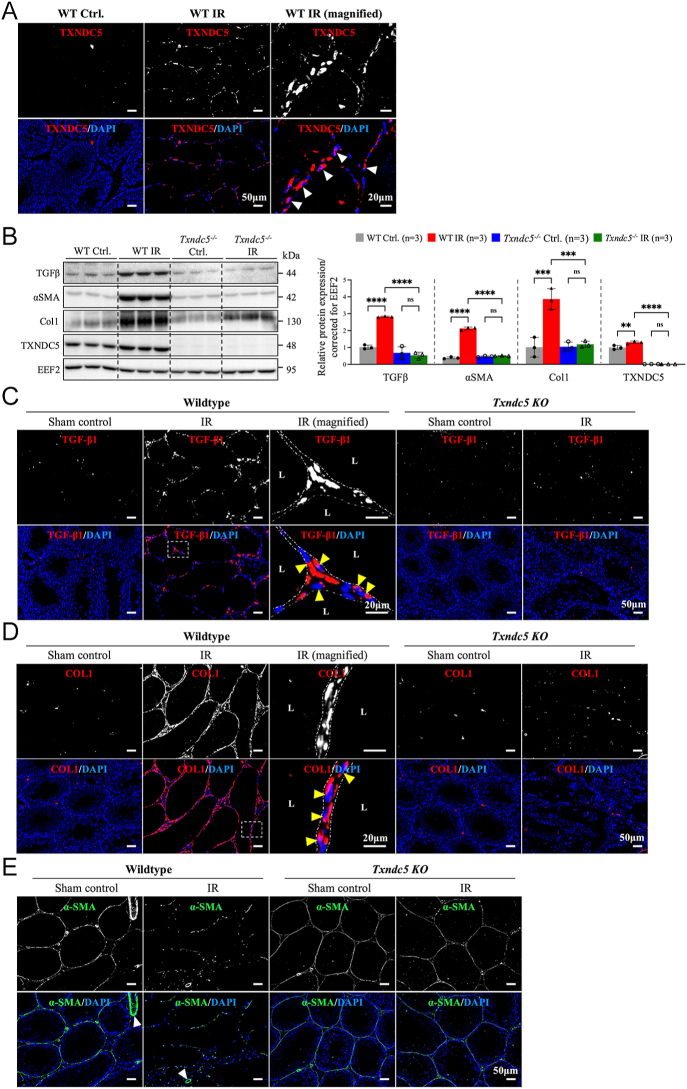
The effects of *Txndc5* KO on IR-induced testicular fibrosis. (A) Upregulation of TXNDC5 was observed in WT IR testes compared to WT control testes. (B) Quantification analysis supported a significant reduction in the protein expression level of fibrosis-related proteins, including TGFβ, COL1 and αSMA, in the testes of IR-injured *Txndc5^−/−^* mice. (C and E) Immunofluorescence staining of TGFβ, COL1 and αSMA in both WT and *Txndc5^−/−^* mice testicular sections. Cellular localization of TGFβ and COL1 were shown in the magnified images (indicated with arrowheads). One-way analysis of variance (ANOVA) with Tukey’s multiple comparisons test was used for group comparisons and statistical difference at *P* < 0.05 (****P < 0.0001, ***P < 0.001, **P < 0.01, ns not statistically different). L, lumen.

In line with recent findings in pulmonary fibrosis, we observed increased TXNDC5 protein expression along with all major fibrosis-associated proteins (i.e. TGFβ, COL1 and αSMA) in WT control testes (Fig. 4A and B); these abovementioned elevations were substantially reduced in *Txndc5^−/−^* mice (Fig. 4B). Immunofluorescent staining further supported the observation as we observed that in contrast to the apparently increased protein expressions of TGFβ (Fig. 4C, indicated with arrowheads) and COL1 (Fig. 4D, indicated with arrowheads) in WT IR testis, a significant reduction in TGFβ (Fig. 4C) and COL1 (Fig. 4D) signals was detected in *Txndc5^−/−^* IR testis. It is worth mentioning that in contrast to the fragmented peritubular αSMA signal detected in the WT IR testes, the αSMA signal remains intact and continuous surrounding the lumens in *Txndc5^−/−^* IR testes (Fig. 4E).

### RNA-seq revealed distinct transcriptomic profiles between WT control, WT IR, *Txndc5^−/−^* control and *Txndc5^−/−^* IR-treated testes with specific pathways involved in fibrosis and oxidation regulation

**Figure 5:**
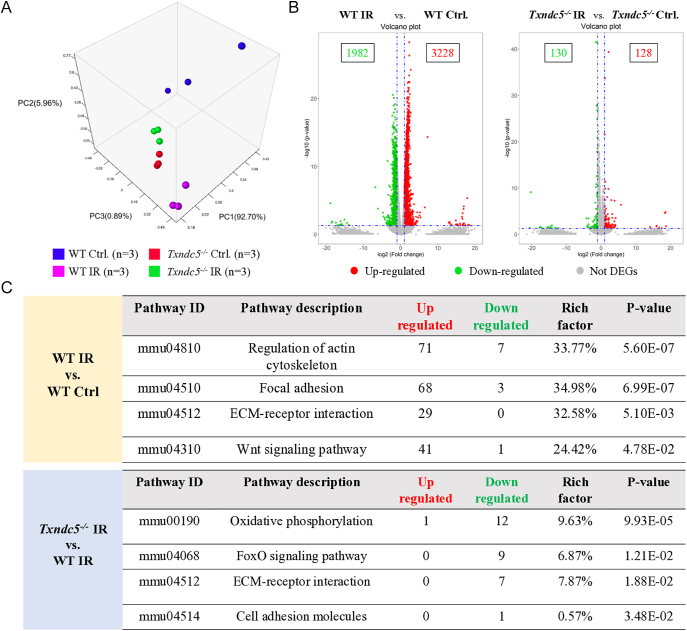
RNA-seq analysis manifested DEGs and signaling pathways affected by IR injury and *Txndc5* deletion. (A) PCA showed distinct overall transcriptomic profile distributions of WT Ctrl, WT IR, *Txndc5^−/−^* Ctrl and *Txndc5^−/−^* IR testes. (B) The volcano plot demonstrated a total of 5,210 DEGs (3,220 up- and 1,982 down-regulated) in WT IR versus WT Ctrl, and 258 DEGs (128 up- and 130 down-regulated) in *Txndc5^−/−^* IR versus *Txndc5^−/−^* Ctrl. (C) Selected signaling pathways with *P*-value <0.05 that were relevant to IR-induced testicular fibrosis.

To investigate the overall gene expression profile between groups (WT Ctrl, WT IR, *Txndc5*^−/−^ Ctrl and *Txndc5*^−/−^ IR), next-generation sequencing was carried out. Three-dimensional principal component analysis (PCA) visualized a distinct gene expression profile within WT Ctrl (blue dots), WT IR (purple dots), *Txndc5*^−/−^ Ctrl (red dots) and *Txndc5*^−/−^ IR (green dots) testes samples (Fig. 5A). Volcano plot revealed abundant DEGs after IR surgery in WT mice with 3,228 upregulated and 1,982 downregulated genes (Fig. 5B). In contrast, we only observed 128 upregulated and 130 downregulated genes upon IR injury in *Txndc5*^−/−^ mice (Fig. 5B). Pathway enrichment analysis identified highly activated signaling pathways (*P*-value <0.05) such as regulation of actin cytoskeleton, focal adhesion, ECM-receptor interaction and Wnt signaling pathway that were relevant to organ fibrosis when compared WT IR with WT Ctrl (Fig. 5C). Moreover, most genes involved in the abovementioned pathways were upregulated. Intriguingly, deletion of *Txndc5*, downregulated genes involved in the regulation of oxidative stress and fibrosis-related pathways, including oxidative phosphorylation, FoxO signaling pathway, ECM-receptor interaction and cell adhesion molecules, indicating the mitigation of oxidative stress and fibrosis-related pathological conditions (Fig. 5C).

### Pathways involved in hypoxia, apoptosis, fibrosis and ECM-receptor interactions were upregulated in IR-injured testes and were downregulated in *Txndc5^−/−^* testes

**Figure 6:**
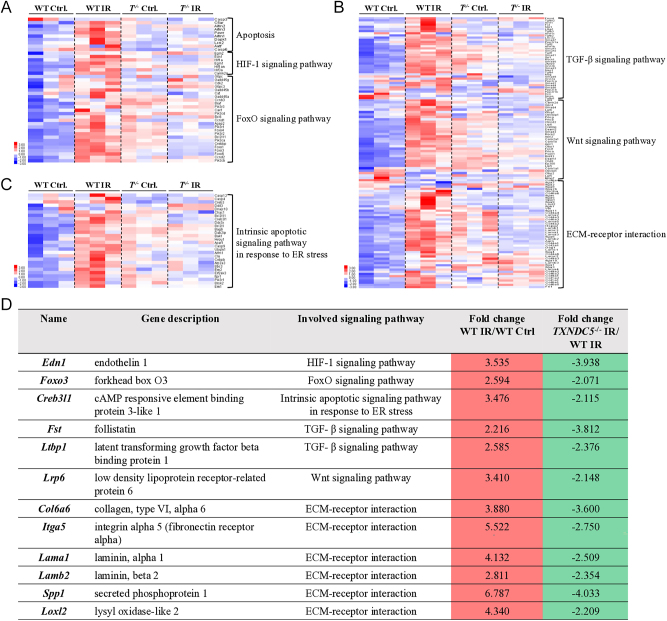
Hierarchical heatmap showed the up- and downregulation of fibrosis-relevant signaling pathways. The expression pattern of genes involved in (A) oxidative stress, (B) fibrosis and (C) ER stress-related signaling pathways. (D) Selected DEGs from the above signaling pathways, which showed upregulation when compared between WT IR/WT Ctrl and downregulation upon *Txndc5^−/−^* IR/WT IR comparison.

Hierarchically clustered heatmap analysis visualized the expression patterns of upregulated and downregulated genes in WT and *Txndc5^−/−^* mice with or without the induction of testicular fibrosis. Upon IR injury, genes involved in apoptosis, hypoxia-inducible factor 1 (HIF-1) (Zhang *et al.* 2021), and forehead box O (FoxO) signaling pathway (Zuo *et al.* 2023) that have been reported to participate in the regulation of oxidative stress were upregulated in the WT testis (Fig. 6A). Moreover, the TGFβ signaling pathway (Peng *et al.* 2022), the Wnt signaling pathway (Burgy & Königshoff 2018) and the ECM-receptor interaction, which were major regulators for organ fibrosis, were also upregulated in the WT testis (Fig. 6B). Genes involved in the abovementioned signaling pathways were mostly downregulated in *Txndc5^−/−^* testes upon IR injury. Moreover, emerging evidence has shown that severe or prolonged ER stress promotes the development of fibrotic disorders (Kropski & Blackwell 2018). In agreement with this, genes involved in intrinsic apoptosis signaling pathways in response to ER stress were upregulated in WT IR testes compared to WT Ctrl (Fig. 6C). By knocking out *Txndc5*, those genes were downregulated in *Txndc5^−/−^* IR compared to WT IR, manifesting alleviation of ER stress and testicular damage in *Txndc5^−/−^* testes.

### Identification of crucial DEGs that might be involved in the TXNDC5-associated alleviation of IR-induced testicular fibrosis

To identify genes upregulated upon IR injury but downregulated upon *Txndc5* deletion, we further selected the overlapped DEGs from the abovementioned active signaling pathways (Fig. 6D). Upon hypoxia conditions, overexpression of endothelin 1 (*Edn1*) was known to mediate by HIF in endothelium and vascular smooth muscle cells (Li *et al.* 2012). Other transcription factors that were known to facilitate stress responses, such as forkhead box O3 (*Foxo3*) (Bernardo *et al.* 2023) and cAMP-responsive element-binding protein 3-like 1 (*Creb3l1*) (Yan *et al.* 2022), were also among the list of DEGs. Follistatin (*Fst*), a profibrotic glycoprotein (Zheng *et al.* 2017), low-density lipoprotein receptor-related protein 6 (*Lrp6*), a transmembrane protein that interacts with Wnt ligands (Yu *et al.* 2020) and ECM-receptor interaction-associated genes such as collagen type VI alpha 6 (*Col6a6*), integrin alpha 5 (*Itga5*), laminin alpha 1 (*Lama1*), laminin beta 2 (*Lamb2*), secreted phosphoprotein 1 (*Spp1*) (Ding *et al.* 2024) and lysyl oxidase-like 2 (*Loxl2*) (Ikenaga *et al.* 2017) were all identified based on the abovementioned criteria, supported the fact that *Txndc5* deletion could ameliorate the genes and signaling pathways activated by IR-induced testicular fibrosis.

## Discussion

The ER is a cellular organelle critical for calcium storage, stress response, protein synthesis, folding and maturation (Schwarz & Blower 2016). ER stress occurs when cells are confronted by harsh conditions such as nutrient deprivation, altered calcium homeostasis, cellular hypoxia and free radical exposure. It has been shown that on torsion/detorsion-induced testicular injury, ER stress-associated molecules such as eukaryotic translation initiation factor 2 subunit alpha (EIF2α) and CCAAT/enhancer-binding protein homologous protein (CHOP) were upregulated, suggesting the involvement in ER physiology in oxidative stress-induced testicular injury (Huang *et al.* 2012). Upon ER stress, dysfunction in protein folding and quality control are common features that lead to fibrotic diseases characterized by extensive production of ECM proteins and organ impairment (Kropski & Blackwell 2018). Previous research has demonstrated that ER protein TXNDC5 promotes fibrosis in myocardial (Shih *et al.* 2018), pulmonary (Lee *et al.* 2020), renal (Chen *et al.* 2021) and liver (Hung *et al.* 2022*a*) fibrosis by facilitating TGFβ signaling, ECM protein folding and stabilizing pro-fibrotic proteins. While deletion of *Txndc5* ameliorates fibrosis progression by inhibiting the activation of αSMA-expressing myofibroblasts and the production of fibrogenic proteins (Hung *et al.* 2022*b*). In agreement with those studies, we observed TXNDC5 upregulation in our IR-induced testicular fibrosis mouse model and increased TGFβ, αSMA and collagen type 1 protein expression. More importantly, deletion of the *Txndc5* gene not only attenuated the severity of testicular fibrosis but the recovery of testicular structure and spermatogenic cell layers with reduced TGFβ, αSMA and collagen type 1 were all detected.

Fibrosis is characterized by overwhelmed collagen accumulation resulting from an imbalance between augmented synthesis and impaired degradation of ECM components during tissue repair and remodeling (Duarte *et al.* 2015). In skin fibroblast from scleroderma patients, nuclear localization of active SMAD3 with elevated *TGFβ* gene expression was observed (Mori *et al.* 2003). In addition, the SMAD3/SP1 protein complex transactivated the promoter activity of collagen type 1 alpha 2 (COL1A2) in human mesangial cells (Poncelet & Schnaper 2001), highlighting the essential role of SMAD3 in regulating TGFβ-induced ECM deposition. In our transcriptomic analysis, *Smad3* was significantly upregulated in WT mice after IR injury and was downregulated in *Txndc5^*−/−*^* IR mice. Besides the TGFβ signaling pathway, emerging evidence has shown the importance of the Wnt signaling pathway in wound healing and fibrosis processes (Burgy & Königshoff 2018). Earlier studies demonstrated that uncontrolled activation of the Wnt/β-catenin pathway led to idiopathic pulmonary fibrosis (Königshoff *et al.* 2008), renal interstitial fibrosis (He *et al.* 2009) and dermal fibrosis (Wei *et al.* 2011). Another study also showed that activation of the Wnt signaling pathway is necessary for TGFβ-induced fibrogenesis (Akhmetshina *et al.* 2012). Moreover, overexpression of Wnt/β-catenin in spermatogenic cells resulted in defective spermatogenesis, revealing the essential balanced Wnt signaling in male germ cell development (Kumar *et al.* 2016). This may explain the absence of sperm cells after IR and the recovery of spermatogenesis upon *Txndc5* deletion.

Low-density lipoprotein receptor-related protein receptor 6 (*Lrp6*), which was identified as a DEG in our model and attenuated upon deletion of *Txndc5*, served as the receptor for Wnt proteins. Upon ligand–receptor binding, downstream β-catenin induces the transcription of Wnt target genes (Nusse 2005). Based on our data, besides the previously known TGFβ signaling pathway, genes involved in both Wnt and ECM signaling pathways were downregulated upon the deletion of *Txndc5*, suggesting that both TGFβ and Wnt fibrogenic signaling pathways were deactivated in our IR mouse model.

## Conclusion

**Figure 7:**
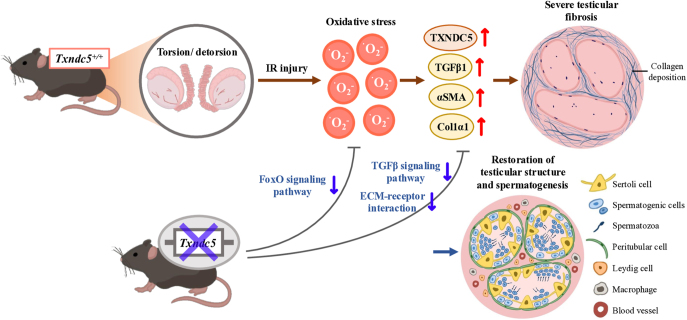
Schematic illustration of IR-induced testicular fibrosis and the recovery upon *Txndc5* deletion.

In conclusion, we have demonstrated in our present study that TXNDC5 plays an essential role in IR-induced testicular fibrosis. Upon IR injury, boosted ROS triggered oxidative stress and pro-fibrotic cytokine TGFβ that subsequently upregulated TXNDC5, which, in turn, activates αSMA^+^ myofibroblast differentiation and proper ECM protein folding. Disrupted testicular architecture and the loss of spermatogenic cell layers were restored in *Txndc5* knockout mice with reduced fibrosis-related protein expressions (Fig. 7). The transcriptomic analysis further confirmed that genes involved in oxidative stress, ER stress and fibrosis-relevant signaling pathways were upregulated in IR-induced testicular fibrosis but were downregulated after the deletion of *Txndc5* in mice. Our results not only demonstrated the essential importance of TXNDC5 in the regulation of oxidative stress-induced testicular fibrosis but also implied the therapeutic potential of TXNDC5 for testicular torsion patients to prevent the development of testicular fibrosis and the restoration of fertility.

## Supplementary materials



## Declaration of interest

The authors declare that there is no conflict of interest that could be perceived as prejudicing the impartiality of the work reported.

## Funding

This study was financially supported by the National Science Councilhttps://doi.org/10.13039/501100001868, Taiwan (grant# NSTC 113-2313-002-006-MY3), and by the Ministry of Educationhttps://doi.org/10.13039/100010002, Taiwan (grant# NTU-114L894203).

## Author contribution statement

YY performed data collection, data analysis, data interpretation and writing. YM helped with methodology and resources. KC contributed to project management and editing. PS contributed to conceptualization, resources, funding acquisition, supervision and editing. All authors have reviewed and approved the manuscript for publication.
