# Supplementary material for: Deletion of TXNDC5 downregulates TGFβ1-αSMA-mediated testicular fibrosis in mice
Source: Reproduction. 2025 May 2;169(6):e250022. doi: 10.1530/REP-25-0022 (PMC12060786; doi:10.1530/REP-25-0022)
Supplement: Supplementary file 1 [file supplementary_materials.pdf]

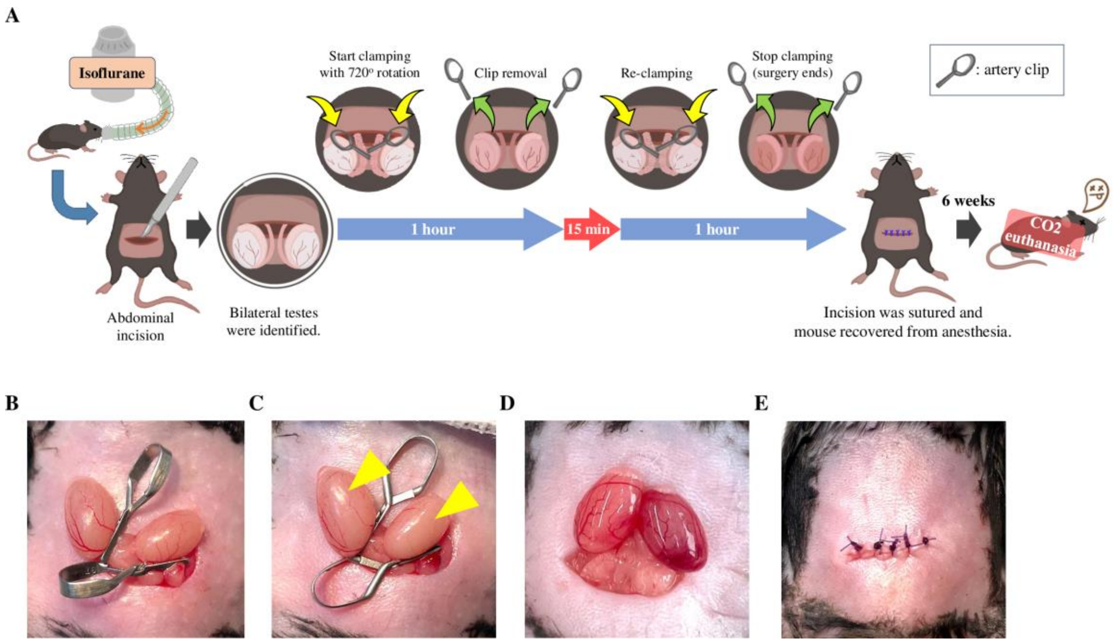

Supplementary Fig 1. Surgical procedure of testicular torsion/detorsion to induce ischemia/reperfusion injury. (A) Testicular IR surgery was carried out by 2 hours of ischemia with an interval of 15 minutes of reperfusion period. Mice were recovered from general anesthesia and underwent carbon dioxide euthanasia for testes sample collection 6 weeks after the surgery. (B) Blood vessels were visible on the surface of the testes in the beginning of the surgery. (C) Disappearance of part of the blood vessels was observed around 30 minutes after testicular arteries clamped by sterilized artery clips. (D) Upon removal of artery clips and rotating the testis to its original position, reperfusion was confirmed by visualization of a hyperemia response. (E) Abdominal incision was closed by suturing the layers.

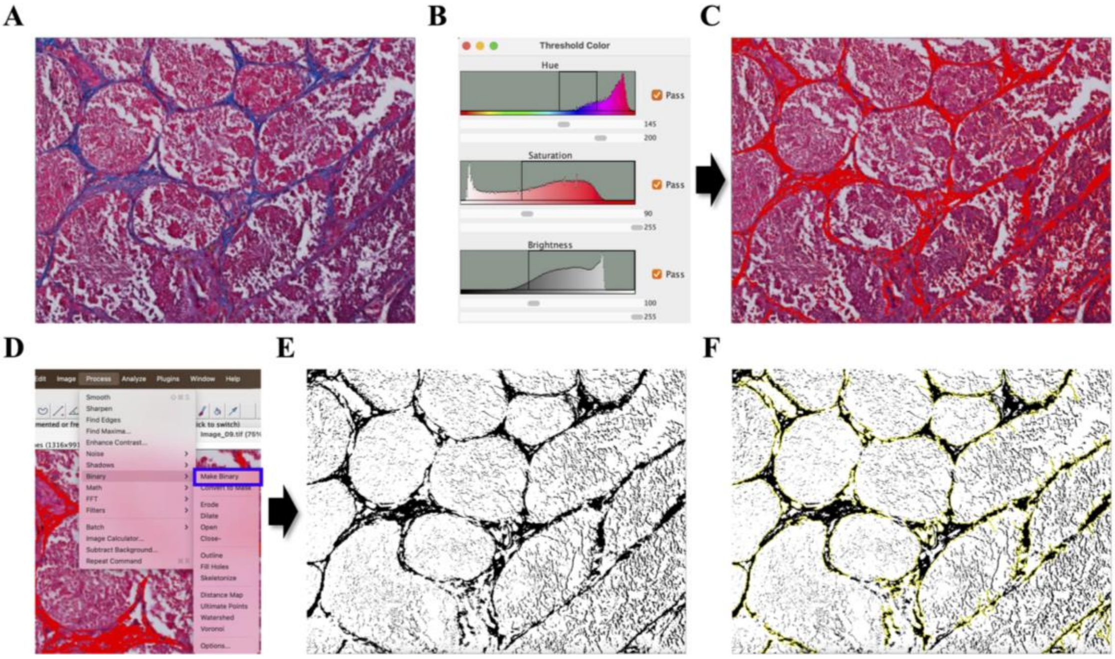

Supplementary Fig 2. Quantification of fibrosis area (%) in Masson's trichrome images. (A) The image was first opened with ImageJ software. (B) (C) Under the "Image" menu, select "Adjust" then "Color Threshold." The hue (145-200), saturation (90-255), and brightness (100-255) were set at these values to achieve optimal blue color intensity. (D) (E) Under the "Process" menu, click "Binary" then "Make Binary"; a greyscale image was achieved. (F) Collagen-positive areas were selected, and area fraction (%) was measured.

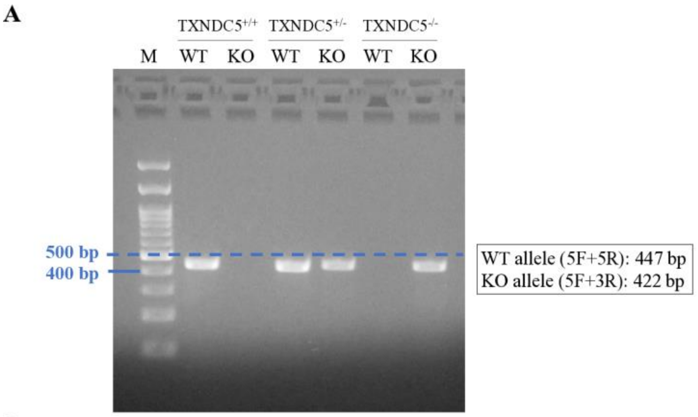

B

5F (common forward)	5'-GGAGGAAGTGATGCCAAACTAGA-3'
5R (WT reverse)	5'-GTTGTACTTGTCTCCCAGGTCAT-3'
3R (KO reverse)	5'-GGATGAGTAATGGAGTCGTGTGT-3'

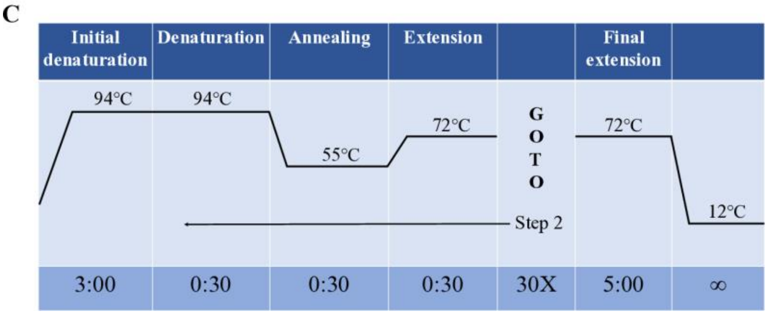

Supplementary Fig 3. Genotyping of WT (*Txndc5*$^{+/+}$), heterozygous (*Txndc5*$^{+/-}$), and knockout (*Txndc5*$^{-/-}$) mice by PCR. (A) PCR result. (B) Primer sets. (C) PCR program.

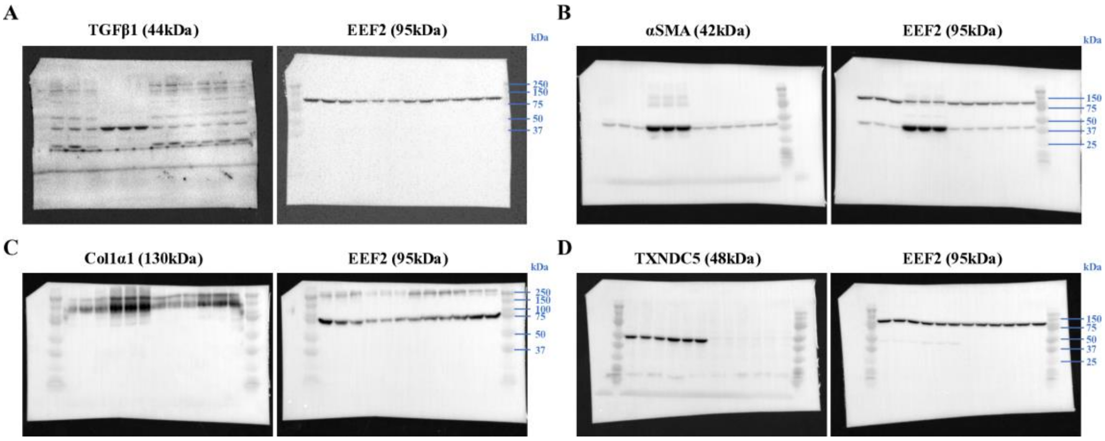

Supplementary Fig 4. Immuno-blotting results of WT and *Txndc5*$^{-/-}$ mice. (A) TGFβ1. (B) αSMA. (C) Col1α1. (D) TXNDC5.
